# I’m still here and my opinion matters: a scoping review on the experience of epistemic injustice among people living with dementia

**DOI:** 10.1007/s12144-025-08519-y

**Published:** 2025-12-17

**Authors:** Lara Calabrese, Marco Brigiano, Martina Quartarone, Ilaria Chirico, Sara Trolese, Francesca Lambiase, Ludovica Forte, Alice Annini, Lisa Bortolotti, Rabih Chattat

**Affiliations:** 1 Department of Psychology, University of Bologna, Bologna, Italy; 2 Department of Philosophy, University of Birmingham, Birmingham, UK; 3 Department of Neurosciences and Rehabilitation, University of Ferrara, Ferrara, Italy

**Keywords:** Epistemic injustice, Dementia, Knowledge, Participation, Scoping review

## Abstract

Epistemic injustice refers to wrongs done to individuals in their capacity as knowers, often due to prejudice or stereotypes. People living with dementia (PLWD) are particularly vulnerable to epistemic injustice due to the cognitive, emotional and social aspects of their condition and this could negatively affect their quality of life. This scoping review aims to map the available evidence on how epistemic injustice can influence the experience of PLWD. By adopting the PRISMA and Joanna Briggs Institute guidance for scoping reviews, we included peer-reviewed and grey literature in English that describe the relation between the presence of epistemic injustice (*concept*) and the experience of PLWD (*population*) across different geographical and cultural contexts (*context*). Searches in academic databases (Web of Science, Proquest, PubMed, Scopus and EbscoHost) and among grey literature (OpenAlex and AlmaStart Discovery Tool) were conducted in November 2024. Two independent reviewers screened abstracts and full texts. A thematic analysis of the results was carried out. We included 10 studies, of which 7 from database searches, 2 from grey literature and 1 from reference lists of included studies. There was high methodological heterogeneity but most of the included studies were theoretical reflections. PLWD can experience epistemic injustice and are often excluded from clinical interactions and academic research due to communication challenges and difficulties in obtaining informed consent. Even when included in clinical practice and research, their voices are often not valued, limiting their involvement in decisions like advance directives, reinforcing negative stereotypes.

## Introduction

### Background

The umbrella term *dementia* refers to a major neurocognitive disorder characterized by a decline in typical cognitive functions, including impairments in information processing, language abilities, and memory (Spencer, 2023). These disease-related characteristics make people living with dementia one of the populations most at risk of being marginalised and excluded from knowledge and decision-making processes (Young et al., 2019). It is important to focus on this type of population because it is constantly increasing worldwide: according to the World Alzheimer Report (2024), it is estimated that over 55 million people currently live with dementia, a number expected to double every 20 years—reaching 78 million in 2030 and 139 million by 2050. As the disease advances, individuals experience a decline in cognitive functions, including the ability to comprehend and produce speech (Aarts et al., 2019). People with late-stage dementia often lose the ability to express themselves verbally due to the progressive damage to areas of the brain responsible for language and communication—particularly the hippocampus, temporal lobes, and frontal cortex, which are critical for memory, speech, and understanding language.

Stigma plays a crucial role in shaping how dementia is socially perceived and how individuals with the condition are treated. It has been defined as a social process through which certain attributes or behaviours are negatively evaluated, leading to the exclusion and discrimination of individuals who embody them (Corrigan et al., 2005). In the context of dementia, stigma encompasses negative stereotypes, prejudices, and discriminatory practices directed not only toward people living with the condition but also toward their caregivers and family members (Brigiano et al., 2025). Dementia-related stereotypes often portray individuals as hopeless, entirely dependent, and cognitively incompetent, with presumed irreversible losses (Young et al., 2019). The literature identifies multiple dimensions of stigma: (1) *public stigma* refers to the endorsement of prejudices, stereotypes, and discriminatory behaviours by the general public toward individuals or groups with a stigmatised condition, such as dementia (Corrigan et al., 2005); (2) *self-stigma* involves the internalisation of societal stereotypes by individuals with the stigmatised condition, as when a person living with dementia may begin to perceive themselves as useless or incapable, reducing self-esteem, increasing shame, and decreasing social engagement (Young et al., 2019); (3) *courtesy stigma* affects those socially or emotionally close to someone with a stigmatised condition, such as caregivers or family members, who may experience discrimination or social avoidance because of this association (Harper et al., 2019; Young et al., 2019).

In this background, the notion of *epistemic injustice* offers a powerful conceptual lens for understanding how social and cognitive exclusion operate in the lives of people living with dementia. The term was introduced by philosopher Miranda Fricker in her book Epistemic Injustice: Power and Ethics of Knowing (2007). The word *epistemic* originates from the Greek *epistēmē*, meaning “knowledge” or “understanding.” It refers to matters concerning belief, justification, and the cognitive processes involved in acquiring and validating knowledge, while *injustice* concerns the violation of a right that causes harm to those whose rights are denied (Oxford English Dictionary, n.d.).

Epistemic injustice is conceptualized as a harm inflicted upon an individual specifically in their capacity as a *knower*, whereby certain people are partially or entirely excluded from processes of knowledge production (Young et al., 2019). This exclusion constitutes an injustice insofar as individuals are denied epistemic credibility due to prejudicial stereotypes associated with the speaker or their social group (Halonen et al., 2024). Fricker (2007) identified two main forms of epistemic injustice: *testimonial injustice* and *hermeneutical injustice*. Testimonial injustice arises when a speaker’s credibility is unjustly deflated due to the listener’s prejudicial biases (Spencer, 2023)—for example, when individuals are unfairly judged as incompetent or unintelligent based on gender, race, or other characteristics (Jongsma et al., 2017). *Hermeneutic injustice* occurs when individuals or groups struggle to communicate their social experiences because society lacks the interpretative frameworks to understand them (Halonen et al., 2024). A classic example is the historical difficulty women encountered in articulating experiences of sexual harassment before the concept was socially recognised (Nathan, 2022).

The term “epistemic injustice” has gained increasing attention in recent years. Although originally developed in philosophy, ethics, and political theory, it has been increasingly applied in the health sciences since 2017—including in medicine, psychology, nursing, neuroscience, and related fields (Baez-Vizcaíno, 2024). The United States (27%), the United Kingdom (23%), and Canada (9%) lead in publications, followed by Australia, Germany, and South Africa (Baez-Vizcaíno, 2024).

Within the dementia field, epistemic injustice offers a valuable framework to understand how stigma and social prejudice translate into systematic exclusion from knowledge production, decision-making, and self-expression. People living with dementia (PLWD) are particularly vulnerable because their cognitive changes are often interpreted as a total loss of epistemic capacity. Being subjected to epistemic injustice can deeply affect quality of life, leading to social withdrawal, isolation, helplessness, and low self-esteem (Young et al., 2019). Beyond the personal level, it also affects society at large, limiting access to diverse perspectives, hindering the development of inclusive responses to social problems, and weakening mutual understanding (Spencer, 2023).

This work seeks to systematically map existing studies on epistemic injustice in the dementia context, aiming to provide a clearer understanding of the specific contexts and mechanisms through which epistemic injustice manifests. It examines both peer-reviewed and grey literature to offer a comprehensive perspective on a phenomenon that remains relatively recent and underexplored. The goal is to raise awareness of an under-investigated issue and highlight the importance of developing interventions to reduce its impact on people living with dementia.

### Review questions

The general objective of this review is to scope and critically synthesise the recent body of literature addressing epistemic injustice in the context of dementia, to identify the types of available evidence concerning its impact on people living with dementia, and to delineate existing knowledge gaps in this emerging field. This work follows established methodological guidance for determining when a scoping rather than a systematic review is most appropriate (Munn et al., 2018).

In particular, this review seeks to elucidate how epistemic injustice—manifesting in testimonial and hermeneutical forms—affects the lived experiences, autonomy, and social participation of people living with dementia. Specific research questions include:

1. How do people living with dementia experience epistemic injustice?
2. What is the impact of epistemic injustice on people living with dementia?

Beyond mapping the current evidence base, this review aims to inform future research and practice directions that could mitigate epistemic injustices and thereby enhance the quality of life, dignity, and agency of people with dementia. By identifying areas where epistemic recognition is lacking, this work also aspires to guide interventions that promote inclusive communication, participatory care practices, and policies that value the experiential knowledge of those with cognitive impairments.

Future objectives include exploring which specific behavioural, institutional, and cultural changes could reduce epistemic marginalisation—such as training for healthcare professionals to recognise testimonial injustice, co-production of knowledge with people living with dementia, and the development of frameworks that ensure their perspectives are systematically integrated into care and research. Ultimately, this review seeks not only to map the state of knowledge but also to contribute to a broader reflection on how epistemic justice can be enacted to foster more equitable dementia care.

## Method

### Design, protocol, and registration

To effectively address our specific research questions, we adopted a scoping review as our chosen method of evidence synthesis. Scoping reviews are particularly suitable for mapping the existing literature, identifying gaps in knowledge, and clarifying concepts (Munn et al., 2018). By utilising this method, we were able to maintain a rigorous and transparent process for searching and synthesising evidence. This scoping review follows the Joanna Briggs Institute’s (JBI) guidance for scoping reviews (Peters et al., 2020) and adheres to the PRISMA extension for Scoping Reviews reporting guidance (Tricco et al., 2018). A scoping review protocol was developed in November 2024 and is available on the Open Science Framework (https://osf.io/vd3aj/). The PRISMA checklist for Scoping Reviews (Tricco et al., 2018) is attached in Supplementary File 1.

### Eligibility criteria

Sources of evidence were assessed for inclusion based on the JBI’s criteria of Population, Concept, and Context (Peters et al., 2020). These criteria were selected to ensure a transparent and systematic approach to determining the relevance of studies addressing epistemic injustice among people living with dementia (PLWD). The rationale for the inclusion and exclusion of studies was guided by the objective of capturing the widest possible range of perspectives and experiences while maintaining conceptual and methodological coherence.

#### Population

We included sources that examined the experiences of people living with dementia (PLWD) across various stages of the condition, as well as studies that compared these experiences with those of other clinical populations, such as individuals with psychiatric diagnoses or those with physical or cognitive disabilities. Including comparative populations was intended to contextualise epistemic injustice in dementia within a broader spectrum of health-related marginalisation, allowing for a more nuanced understanding of which aspects may be unique to dementia and which are shared across different conditions. There were no specific inclusion criteria based on age, gender, or other demographic or qualifying characteristics, as our aim was to represent the diversity of experiences within this population. The only studies excluded were those that did not specifically address the experiences of PLWD, ensuring that the review maintained a focused perspective on this group and their epistemic positioning within care and research contexts.

#### Concept

The central concept explored in this scoping review is epistemic injustice, a term introduced by philosopher Miranda Fricker in 2007. Epistemic injustice refers to the ways in which prejudice and social bias can undermine an individual’s credibility and capacity to contribute to shared knowledge. Such injustices often result in the marginalisation of certain groups within processes of meaning-making and decision-making (Young et al., 2019). Fricker identified two key forms of epistemic injustice: testimonial injustice, which occurs when a person’s word is undervalued due to prejudice, and hermeneutic injustice, which arises when collective interpretative resources are insufficient to adequately represent someone’s experiences. We included studies that addressed one or both of these forms, whether explicitly or implicitly, to capture the breadth of ways epistemic injustice manifests in the lives of PLWD. This conceptual focus allowed us to integrate theoretical and empirical perspectives, supporting a more comprehensive analysis of how epistemic injustice operates in dementia care, research, and everyday life.

#### Context

No exclusion criteria were imposed regarding the context in which the research took place. This decision was made to map the diversity of settings—clinical, community, institutional, and socio-cultural—in which epistemic injustice may occur, and to identify potential disparities in where and how this topic has been explored. By adopting this inclusive approach, we aimed to highlight geographical and contextual gaps in the existing evidence base and to identify underrepresented areas where further research may be particularly valuable. This rationale reflects the exploratory nature of a scoping review and acknowledges that epistemic injustice is shaped by contextual and relational factors that vary across care environments and cultural settings.

## Search strategy

Our searches included peer-reviewed and grey literature in five electronic databases: PubMed, Web of Science, Pro-Quest Central, Scopus and EBSCOhost. The following databases were included in EBSCOhost: APA PsycInfo, APA PsycArticles, CINAHL Complete and Psychology and Behavioral Sciences Collection. Other studies were included by searching the bibliographies of the sources previously included. The search string has been adapted to individual databases, an example of the search string utilized on PubMed is provided in Table 1. A Librarian was consulted for database research and for the developing of the search string. The search string was initially developed based on keywords extracted from informing literature and then optimised with support from the Librarian. Searches of grey literature were carried out online, examples of database utilized are OpenAlex and AlmaStart Discovery Tool. The databases used for grey literature were selected after a consultation with the Librarian. The keywords used for the grey literature search were very similar to those used for the peer-reviewed article search, so ‘epistemic injustice’ or ‘testimonial injustice’ or ‘hermeneutical injustice’ (concept) AND ‘dementia’ or ‘Alzheimer’s disease’ or ‘vascular dementia’ or ‘frontotemporal dementia’ or ‘Lewy body dementia’ (population). The searches took place on 19th November 2024. Searches were limited to sources in English language due to the limited resources available to the research team for translation, but no restrictions were applied in terms of date of publication, geographical area or study design.

Once the searches were completed, the identified records were imported into the Rayyan software (Ouzzani et al., 2016) for removal of duplicates and abstract screening. We employed a three-step screening process with initial remotion of duplicates and a consequent assessment of title and abstract in Rayyan, followed by full-text screening carried out independently by two authors (L.C. and M.B.). Any discrepancies were resolved in discussion with a third reviewer (R.C.) or with other members of the research team. The final included studies were entered in an Excel spreadsheet, documenting key information including authors, year of publication, country, methods, participants, aims and results.

Given the inclusion of both peer-reviewed and grey literature, and of different publication types or study designs, we did not carry out a structured critical appraisal of the evidence. This is in line with JBI’s guidance for scoping review, which indicates that quality assessment of individual sources or of meta-bias may not be possible in scoping reviews given the diversity of sources included and the broad aim of mapping the available literature for an understudied topic (Peters et al., 2020).

### Synthesis of results

A thematic analysis was carried out to outline trends in publications’ key study characteristics based on data extraction. The findings were analysed by identifying thematic areas in the included studies, focusing on the context where the presence of epistemic injustice in people living with dementia can be observed. This categorisation was deemed more meaningful than organising on type of methodology used, as the high heterogeneity across the studies included. The aim of our main analysis was to identify how people living with dementia experience epistemic injustice (question 1) and what is its impact on their lives (question 2).

## Results

### Selection of Sources of Evidence

We retrieved a total of 6929 records from the online databases. We removed 2930 duplicates, leaving 3999 records to screen in stage 1. Of these, 3983 were excluded at stage 1, whereas 16 records moved to stage 2 (full-text screening). At this stage, 9 records were excluded because they did not meet inclusion criteria. Seven of these were excluded due to lack of focus on epistemic injustice (concept) and 2 because they did not focus on epistemic injustice (concept) and did not talk about people living with dementia (population). A total of 7 records from the database search were included in the review.

Beyond the database searches, we identified 5 records from the grey literature from websites and 4 records from the reference lists of included studies. Of these additional 9 records, 6 were excluded during the full text screening: 4 because they did not talk about epistemic injustice and 2 because they did not talk about people living with dementia.

**Fig. 1:**
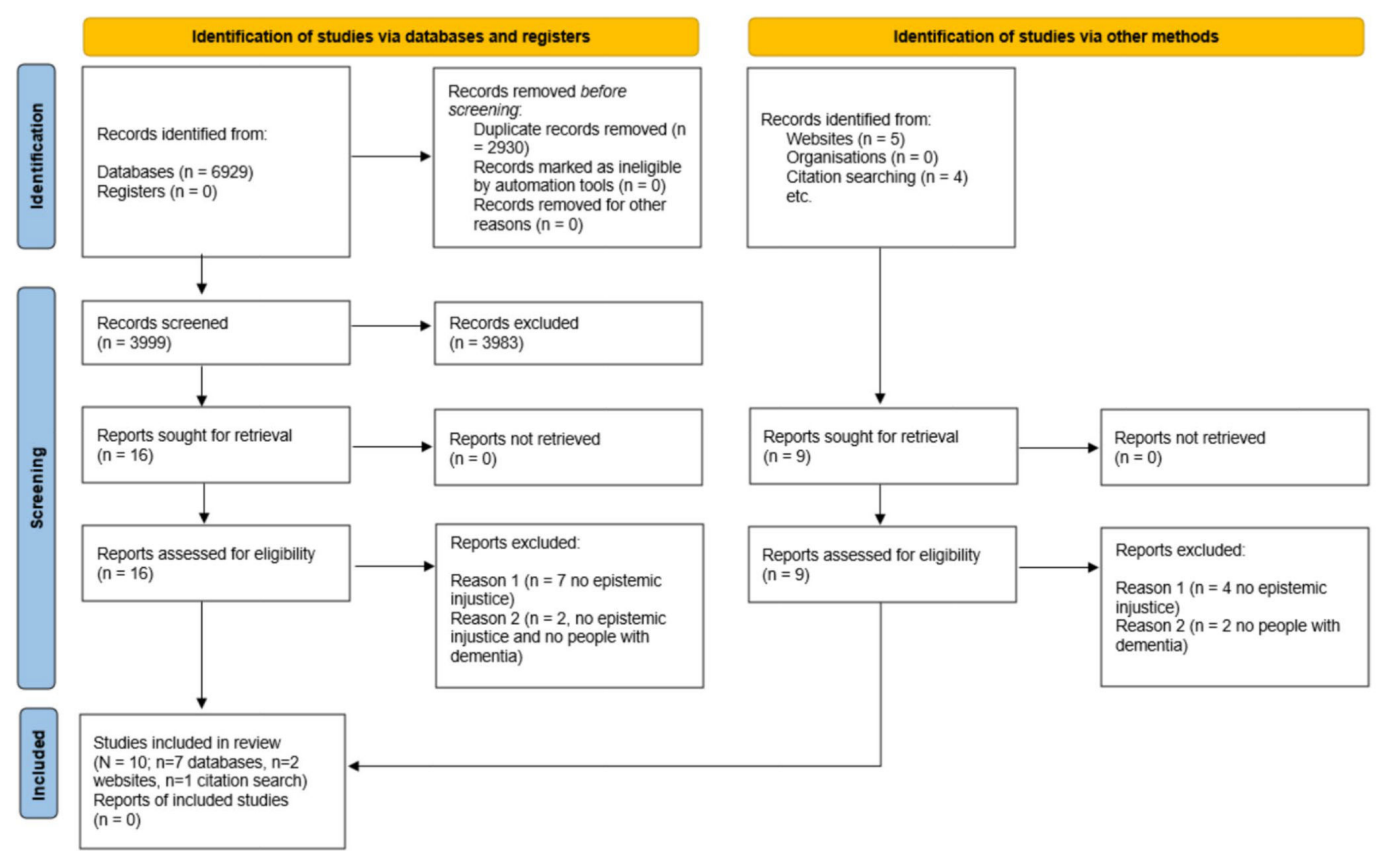
PRISMA 2020 flowchart for the scoping review process (Page et al., 2021)

In summary, a total of 10 records were included, of which 7 were from database searches, 2 from grey literature and 1 from reference lists of included studies. A PRISMA flow diagram outlining the screening and selection process is included in Fig. 1.

### Characteristics of sources of evidence

The main characteristics of the records included in the review are presented in Tab. 2 (peer-reviewed literature) and Tab. 3 (grey literature). All the records included were published in the last 10 years. In terms of country distribution, 7 out of 10 studies were conducted in Europe, of which 2 in the United Kingdom, 2 in Germany, 1 in Belgium, 1 in Finland and 1 in the Netherlands. One study was conducted in the USA. Two studies were conducted in multiple countries: 1 between Australia and Canada and 1 between Australia and Scotland.

Regarding study methodologies, all the included studies used a qualitative method (n = 10). Most of the included studies (n = 7) are theoretical reflections, while the remaining are case studies (n = 1), interviews (n = 1) or interviews mixed with focus groups (n = 1). Publication types included empirical peer-reviewed articles (n = 8) and book chapters (n = 2).

Often the studies included were the result of collaboration between researchers from different disciplines (e.g., social health, psychology, philosophy). This contributed to the great methodological variety with which the topic was treated.

### Extracting and charting of the results

Seven of the 10 studies included are theoretical reflections which reflect on the consequences that epistemic injustice may have on the experience of PLWD. In the remaining three articles were carried out with interviews (Jongsma et al., 2017), case studies (Groot et al., 2023), or focus group interviews (Matthews, 2016) with people or researchers working with people living with dementia. None of the included articles carried out interviews or other types of study actively involving people with dementia. One of the included articles (Halonen et al., 2024) mentioned a proposal to interview people living with dementia and their caregivers, but the study has not been published yet. All articles focus on the experience of people with dementia, but in one study (Jongsma et al., 2017) both people with dementia and people with autism were interviewed. The results of the study have not been differentiated for the two different populations because the authors state that it is not their objective to compare the two, but we still felt it was important to include the study in the review because of the relevance of the information gathered in the interviews.

#### Theoretical models and prospectives

Young et al. (2019) highlight several ways in which epistemic injustice can affect people living with dementia (PLWD). They note that such injustice can occur when negative stereotypes about the reduced credibility of PLWD are internalized—not only by healthcare professionals, family members, and friends, but also by the individuals themselves and figures of authority. These stereotypes can take various forms and are reinforced through language, metaphors, dominant medical frameworks, intersecting social biases, and prevailing cultural beliefs. Credibility assessments are often shaped by assumptions about the person’s future cognitive decline, with the expectation that their ability to contribute meaningfully to knowledge production and exchange will diminish over time. When these stereotypes are internalized by others, they may lead to the exclusion of PLWD from epistemic practices, such as being heard, believed, or included in decision-making. Conversely, when they are internalized by PLWD themselves, these stereotypes may result in self-exclusion, as individuals begin to withdraw from participation in knowledge-sharing or communicative practices. In developing the extended self-stigmatization model, Price and Hill (2021) suggest that people living with dementia who perceive their stigmatized condition as legitimate are more likely to experience declines in self-esteem and self-efficacy. This trajectory makes them particularly vulnerable to both externally imposed and self-directed forms of epistemic injustice. As a result, individuals may progressively withdraw from communicative and social engagement, eventually facing what the authors describe as a form of *social death*—where the person is treated as if they no longer exist or are regarded as a ‘non-person’ by those around them (Price & Hill, 2021). Chattat et al. (2024) highlights how the presence of epistemic injustice in the context of dementia can have an influence on social health and consequently on social well-being. Ageism and stigma represent significant barriers to the societal participation of older adults, particularly those with dementia. Discrimination affects various practices, including diagnosis disclosure, care planning, and decision-making involvement, often marginalizing people with dementia (Chattat et al., 2024). Labelling their behaviour of PLWD as a disorder or as a symptom of a disorder disregards the social context and relationships that shape their actions. As a result, individuals with dementia experience epistemic injustice, being excluded from decision-making processes and denied full societal membership (Chattat et al., 2024).

In her paper, Spencer (2023) argues that the concept of testimonial injustice is too restrictive, limiting the phenomenon to cases where people speak and are not listened. Thus, testimonial injustice focuses on “telling” and excludes non-verbal communicative practices. This is a problem because meaningful communication can occur through non-verbal expression. The social perception that recognizes individuals as “knowers” is not confined to speech but also includes bodily behaviour, as meaningful communication can occur non-verbally. Spencer (2023) proposes the concept of *non-verbal testimonial injustice* by expanding the notion of “testimonial sensibility” to include “communicative sensibility” and comprehend all forms of expression, even from those who cannot communicate verbally, for example people with language-affecting dementia. Therefore, even people who are unable to communicate verbally, such as people at late-stage dementia, can be considered victims of testimonial injustice because it is also possible to ‘testify’ nonverbally.

#### Exclusion from participation in research

The study of Halonen et al. (2024) reflects on what enables or prevents people living with dementia from participating in research and how this is connected to epistemic injustice. Researchers play a significant role in the exclusion of people living with dementia from research. Greater efforts are needed to foster inclusive practices by encouraging researchers to act with greater confidence, trust in the value of inclusion, and actively create opportunities for the meaningful participation of individuals with dementia in studies that directly concern them (Halonen et al., 2024). Groot et al. (2023) highlight the idea that the correct way of doing research (that is, following procedures) can also become the wrong way in the study of epistemic injustice because following procedures often involves excluding from participation in research those people who are regarded as unable to provide informed consent. Some researchers prioritize academic objectives without reflecting on whether their work has had a positive impact on the individuals they have studied or whose behaviour they have observed (Groot et al., 2023). Conducting research with PLWD presents important challenges, particularly due to the difficulties some individuals encounter in expressing themselves verbally. As Groot et al. (2023) emphasize, this highlights the need to pay close attention to non-verbal communication to avoid perpetuating epistemic injustice within the research process. The authors also draw attention to the broader issue of the limits of understanding another person. A central concern for researchers is the uncertainty involved in interpreting participants’ behaviours and expressions—a challenge made more complex by the researcher’s active role in translating these non-verbal interactions into academic narratives for publication (Groot et al., 2023). Groot et al. (2023) highlight the importance of evaluating the appropriateness of researchers’ approach to research on an ongoing basis, trying to balance between intuitive and rational forms of interaction and interpretation of communication.

Instead, the study by Halonen et al. (2024) reflects on the two main reasons why people with dementia are excluded from participating in research: the role of gatekeepers and the process of applying for ethical approval. It is often due to the strict criteria imposed by ethics committees that people with dementia are excluded. This contributes to epistemic injustice, because involving people with dementia in research means giving them a voice. Participation in research enable PLWD to contribute to the creation of knowledge about their experience with the disease. Halonen et al. (2024) highlight the need to take a critical look at current inclusion criteria in academic research and encourage a discussion on how people living with dementia can be more actively involved in research, in ways that support both meaningful participation and high-quality research. For example, in research the use of the Mini-Mental State Examination (MMSE) scores, a brief and standardized test used to assess cognitive function and screen for cognitive impairment including dementia, could be problematic and harmful because focuses on weaknesses (Halonen et al., 2024). Even a person with a low score on the MMSE might be able to participate in research, perhaps by being interviewed or engaging in a free conversation. Another form of epistemic injustice in research with PLWD is the inclusion of only people at early stages of dementia or who able to give informed consent (Halonen et al., 2024). This practice could facilitate the process with ethical committees but could also be considered a form of epistemic injustice because those with moderate and severe dementia are excluded from the research (Halonen et al., 2024). Excluding participants based on the strict application of the informed consent criteria could deny to PLWD the position of informants and experts in their own lives with dementia (Halonen et al., 2024).

#### Distrust in the capacity to know at institutional level

An in-depth analysis of interviews with members and representatives of patient organizations (POs) in the study by Jongsma et al. (2017) reveals that persistent stereotypes continue to limit the inclusion of affected individuals both within the organizations themselves and at the level of health policy. Patient organizations play a crucial political role by representing their groups in healthcare policymaking and advocating for the allocation of resources, access to diagnostics, treatment, and care, as well as addressing broader collective concerns such as ethical and legal issues related to research policy, social welfare, and end-of-life rights (Jongsma et al., 2017). Jongsma et al. (2017) report that the extent to which a person is affected by the condition can cause distrust in their *capacity to know* in a two-fold way: it is assumed that those who can represent themselves are “not affected enough” by the condition to present valuable insight into its severity, and those who have difficulties in expressing themselves are “too affected” by the condition and are thus excluded for this reason. The involvement and voice of affected people is sufficiently safeguarded, but this does not necessarily guarantee that they are considered credible because of the several prejudicial stereotypes by the affected members and by most representatives (Jongsma et al., 2017). The authors state that this could be considered as a form of testimonial injustice: “I listen to your opinion, but I do not value it”. The impact of stereotypes and prejudices often culminates in paradoxical reasoning regarding the testimonies of affected individuals who can express their experiences. They are deemed “atypical” or “insufficiently affected” to offer valuable insights into their condition or what benefits the collective, while those considered “truly affected” are perceived as “too affected” to be credible or knowledgeable speakers, resulting in their being “silenced” (Jongsma et al., 2017).

#### Advance directives and ethical implications of epistemic injustice

The presence of epistemic injustice in PLWD has implications on their ethical rights, particularly relevant in this field is the topic observed in the paper of Vulliermet and Kenis (2024): advance directives. Advance directives have gradually become an important instrument that enables individuals to ensure their wishes are respected in situations where they may no longer be able to express them (Vulliermet & Kenis, 2024). The authors assume that the dominant discourse frames dementia as ‘monstrous’, an ‘enemy’, a destructive experience is problematic in several respects (Vulliermet & Kenis, 2024). The predominance of a negative framing of dementia may have significant implications regarding social stigma and the treatment of PLWD, propagating various forms of epistemic injustice (Vulliermet & Kenis, 2024). The question reported by the authors is that the person with dementia, after diagnosis, loses the right to change their opinion about what was previously expressed in their advance directives, meaning that the self with dementia is attributed less importance than the self without dementia. Decisions made by the same person before diagnosis (then self) have more value than decisions made by the current person (now self). Vulliermet and Kenis (2024) identify this phenomenon as epistemic injustice because the person is not given the right to change decisions expressed prior to the diagnosis of dementia, thus their right to make decisions and be an active participant in their care process is denied.

#### Role of media in change representations of dementia: a double-edged sword

In her paper, Matthews (2016) identifies two key factors that contribute to the presence of epistemic injustice in dementia care: (1) affective practices and testimonial sensibilities; and (2) empathy and the pedagogy of discomfort. Dementia training resource, such as the film *Darkness in the Afternoon*, aim to help care workers empathize with PLWD by encouraging them to understand and validate the perspectives of PLWD. These training resources emphasize the importance of not attributing distress solely to the dementia itself but instead considering the broader context of the person’s life story. The training promotes empathy by framing interactions in a way that highlights the value of understanding the emotional experiences of PLWD. Training approaches that involve emotional discomfort, such as the “pedagogy of discomfort”, challenge care workers to confront their own emotional barriers and assumptions about dementia. The training aims to reshape affective practices, fostering a deeper connection and understanding between workers and PLWD. The idea is to push care workers out of their comfort zones, making them reflect on their own emotional responses and biases, which can help them approach PLWD with greater openness and empathy. A key strategy is telling the life stories of PLWD, which humanizes them and fosters a sense of proximity, making workers more attuned to the lived experiences of PLWD. These practices aim to reduce the emotional barriers that contribute to epistemic injustice, where the knowledge and voices of PLWD are often ignored or dismissed (Matthews, 2016).

Capstick et al. (2015) argue that film serves as one of the most powerful tools for change public perceptions of dementia, but the popularization of dementia is rarely neutral or without issues. Portrayals of PLWD in films often rely on negative representations, including total memory loss, violence and aggression, extreme dependence on heroic caregivers, catastrophic outcomes, and early death. Such representations reinforce negative stereotypes, increase the presence of fear and avoidance of dementia, and reaffirms the dominant biomedical view that interprets all actions and words of PLWD solely as symptoms of the disease. Capstick et al. (2015) support the involvement of PLWD in creating educational and awareness-raising materials and advocate the idea that showing participants in a positive light can help reduce the harmful effects of epistemic injustice experienced by PLWD.

## Discussion

The studies included in this scoping review introduce a great variety of contexts where it is possible to observe the occurrence of epistemic injustice. Four studies (Price & Hill, 2021; Spencer, 2023; Young et al., 2019; Anonymized for review et al., 2024) include epistemic injustice within existing theoretical models (Price & Hill, 2021) or reflect on possible practical implications for PLWD. Two studies (Groot et al., 2023; Halonen et al., 2024) have further examined the reasons why PLWD tend to be excluded from participation in academic research. This seems to be associated with the role played by researchers and ethical committees. In the study of Groot et al. (2023) researchers outlined the difficulty of communicating with and understanding PLWD; while the study by Halonen et al. (2024) highlights the importance of including people who are not always able to give their informed consent in participation in research. Both studies mentioned above place the focus on difficulties encountered by researchers, emphasizing how PLWD are excluded from participating in academic research not because they are not able to participate, but because of the problems faced by the researchers. One study (Jongsma et al., 2017) interviewed representatives, members, and policy makers of Patients’ Organization (POs) both in the field of dementia and autism. This study reveals that being heard is not sufficient. Even when individuals with dementia are given the opportunity to speak, their accounts are not necessarily acknowledged as valid contributions or afforded epistemic credibility. This allows us to say that to reduce epistemic injustice, it is not enough to listen to people with dementia but also to recognise the value of their testimonies.

Alzheimer Europe emphasizes the significance of engaging individuals with dementia in research not only as research participants but also through active roles within Patient and Public Involvement (PPI). This includes contributing to the development of research ideas, offering input and guidance to researchers, participating in consultations, and taking part in research activities directly (Gove et al., 2017). Evidence indicates that many people living with dementia can make valuable contributions to research on topics that directly impact their lives (Gove et al., 2017). Their involvement in PPI can be facilitated through diverse methodological approaches, including qualitative, quantitative, mixed-methods, co-production, co-research, and other participatory strategies (Gove et al., 2017). A variety of data collection methods may be used in this context, such as interviews, focus groups, questionnaires, and surveys (Gove et al., 2017). One study (Vulliermet & Kenis, 2024) analyses the implications of the presence of epistemic injustice in advance directives and ethical questions. The dominant negative framing of dementia denies to PLWD the right to take different decisions from those made before being diagnosed with dementia. Two sources (Capstick et al., 2015; Matthews, 2016) investigate the impact of the media in changing the representation of dementia; the authors state that media can have a great impact on how the society see dementia and affected people, so it can be considered a tool in challenging the negative representation of the disease, considering also the point of view of people with dementia. As it transpires from our analysis, the variety of contexts in which the notion of epistemic injustice has been applied to PLWD shows that epistemic injustice affects a number of practices and social interactions.

Young et al. (2019) uses an expression that describes the impact of epistemic injustice on people with dementia: PLWD are *recipients of care*. Epistemic injustice makes people with dementia recipients of care: powerless, passive, dependent, and without communication skills. The consequences of epistemic injustice make relationships with PLWD unidirectional: caregivers are more credible and competent than PLWD. Brooker and Latham (2016) emphasizes the fundamental role of relationships in recognizing that people exist, asserting that interdependence is a necessary condition of being human. Understanding dementia necessitates viewing personhood through relational dynamics. Even when cognitive impairment is very severe, some form of encounter and communication is often possible (Brooker & Latham, 2016; Kitwood, 1997). He introduced the person-centred approach, which focuses on providing care and services that are tailored to the individual and their specific needs. However, in recent years, there has been growing recognition of the need to move beyond this perspective (Nolan et al., 2006). One of the main criticisms of the person-centred model is that it can overlook the importance of reciprocity in care relationships (Nolan et al., 2004). This is especially relevant in care home settings, where relationships between caregivers and residents often develop over long periods of time. In these environments, the most successful care homes function as true “communities,” where there is a strong sense of mutual dependence between those receiving care and those providing it. The idea of relationship-centred care (RCC) was first introduced by Tresolini (1994) and the Pew-Fetzer Task Force in 1994, following a detailed review of the limitations of modern healthcare systems. RCC emphasizes the importance of meeting the needs of everyone involved in the care process, creating a more balanced and connected approach.

Epistemic injustice can be observed also between the elements that contribute to the creation of what Kitwood (1997) called ‘malignant social psychology’ (i.e., disempowerment, banishment, objectification). The term ‘malignant’ does not imply bad intentions from the caregivers where on the contrary much of the work is done with kindness and good intentions. What is malignant is part of our cultural identity (Brooker & Latham, 2016; Kitwood, 1997). Similarly, epistemic injustice is not perpetrated malignantly or intentionally but is part of our cultural identity according to which PLWD are unable to express their thoughts and to contribute to the construction of knowledge. The limited number of articles on epistemic injustice in dementia confirms that this is a very recent topic and requires further exploration in the future. To synthesise, among people living with dementia, epistemic injustice manifests through multiple and interrelated situations. These include being excluded from conversations about their care, having their experience and emotions dismissed as unreliable, being positioned as passive recipients rather than active participants, and being denied recognition of their authority in decision-making processes (Chattat et al., 2024). Such dynamics do not merely reflect isolated episodes but are deeply embedded within the routines of dementia care. Their impact is visible across key stages of the care pathway, including diagnosis disclosure, the process of advance care planning, the practices surrounding shared decision-making, and the interpretation of behavioural and psychological symptoms of dementia (BPSD) (Chattat et al., 2024).

An example of epistemic injustice occurs in the communication of the diagnosis. Empirical studies have shown that, in 66% of cases the diagnosis of dementia is not disclosed directly to the person affected, but rather to their family caregivers (Low et al., 2019). This decision is often shaped by the preferences of caregivers or by clinicians, while PLWD are frequently excluded from the decision-making process. The reasons behind non-disclosure can be traced to two main domains: on the one hand, clinicians may lack adequate training, perceive little benefit in delivering the diagnosis, or are concermed about the emotional repercussions for the patient or family (Chattat et al., 2024). On the other hand, families may fear the psychological impact of the diagnosis or assume that the person lacks the capacity to comprehend the information (Wollney et al., 2022). Despite the ethical implications of this practice, there is a gap in research regarding how people with dementia experience the act of being informed—or not informed—about their condition (Chattat et al., 2024). This gap further reinforces their marginalisation and epistemic vulnerability.

Similar mechanisms can be identified in the context of advance care planning. This process is intended to support individuals in articulating their values, preferences, and goals regarding future care while they are still cognitively able to do so. However, PLWD are frequently excluded from this process, despite having the legal and moral right to participate (Vulliermet & Kenis, 2024). The barriers to their involvement are multifaceted. Professionals often avoid discussing the future trajectory of the disease, either due to discomfort or perceived uselessness (Chattat et al., 2024). Prejudices about the ability of PLWD to engage in complex discussions persist, and there is frequently no structured process to facilitate their gradual acceptance of the diagnosis and the planning that should accompany it.

The issue of shared decision-making further illustrates the epistemic marginalisation of PLWD. In many care settings, individuals are not meaningfully involved in decisions affecting their daily lives—not only in relation to major medical or legal matters, but also in everyday choices such as what to eat or what to wear. These decisions, while seemingly trivial, are central to preserving personal identity and dignity. Yet, various obstacles hinder the implementation of shared decision-making. Professional caregivers and family members often make assumptions about the person’s cognitive limitations, leading them to make choices on their behalf. Additionally, professionals frequently lack training in communication strategies tailored to people with cognitive impairments (Mariani et al., 2017).

Finally, the domain of behavioural and psychological symptoms of dementia (BPSD) offers another example of epistemic injustice. Traditionally, symptoms such as delusions, hallucinations, agitation, anxiety, or changes in sleep and eating patterns are interpreted as neuropsychiatric manifestations of brain pathology requiring control or suppression (Chattat et al., 2024). This biomedical model tends to view such behaviours as meaningless and disruptive. In contrast, the person-centred approach offers an alternative framework, suggesting that these behaviours are, in fact, meaningful expressions of emotional needs, environmental stressors, or unmet psychological or social concerns. This model recognises the communicative intent behind behavioural symptoms and highlights the importance of interpreting them within the relational and environmental context in which they occur. From this perspective, PLWD are no longer passive objects of clinical management, but active participants whose expressions deserve to be heard, understood, and respected. Overall, the variety of contexts in which epistemic injustice permeates multiple aspects of dementia care confirms that it is an extensive phenomenon that requires attention and awareness to decrease its presence. Whether through exclusion from dialogue, denial of credibility, or failure to support agency, people living with dementia often find themselves on the margins of the very systems designed to support them. Addressing these injustices requires not only structural changes in healthcare practices and policy, but also a profound cultural shift in how we understand and value the knowledge and experiences of PLWD.

## Practical implications and recommendations

Although this scoping review primarily aimed to map the existing evidence on epistemic injustice in dementia, the findings also suggest several directions for practical action. Addressing epistemic injustice requires interventions across clinical practice, policy, and education, with the overarching goal of recognising people living with dementia (PLWD) as legitimate knowers and active participants in their own care and in society.

## Clinical practice

In clinical settings, fostering epistemic justice involves promoting communication practices that value and integrate the perspectives of PLWD. Clinicians should be supported in developing relational and communication skills that enable them to interpret expressions, emotions, and behaviours as meaningful rather than merely symptomatic. Shared decision-making models should be systematically implemented, ensuring that PLWD are included in discussions about diagnosis disclosure, care planning, and treatment choices. Routine clinical procedures—such as diagnostic conversations or the management of behavioural and psychological symptoms—should be reframed as opportunities for reciprocal understanding and co-construction of meaning, rather than as unidirectional acts of information delivery.

## Policy

At the policy level, reducing epistemic injustice requires embedding the principle of epistemic inclusion within the design and evaluation of dementia care systems. Health and research policies should promote the participation of PLWD in all stages of research, from agenda setting to dissemination, for example through Patient and Public Involvement (PPI) frameworks. Ethical guidelines and research governance structures should be revised to facilitate the engagement of PLWD in research—even in cases where obtaining traditional informed consent may be challenging. Furthermore, public health strategies should encourage media representations and social narratives that portray dementia in diverse and non-stigmatising ways, thus challenging cultural assumptions that contribute to epistemic exclusion.

## Education and training

Educational initiatives for healthcare professionals, caregivers, and students should explicitly address the concept of epistemic injustice and its implications for dementia care. Training programmes should include content on recognising and mitigating testimonial and hermeneutic injustices, with a focus on empathy, reflective practice, and the relational nature of knowledge. Creating opportunities for professionals to learn directly from people with dementia—for example through participatory workshops or narrative-based education—can help cultivate a more inclusive epistemic culture. Similarly, public education campaigns and community-based initiatives can play a role in reshaping social perceptions of dementia, reinforcing the view that people living with dementia remain capable of meaningful contribution and knowledge sharing.

Overall, promoting epistemic justice in dementia care involves more than ethical commitment; it requires systemic change and a shift in collective mindset. Integrating these recommendations into practice, policy, and education could help transform the experiences of PLWD—from being positioned as passive recipients of care to being recognised as valued contributors to shared understanding and decision-making.

## Knowledge gaps

Most of the studies, both in peer-reviewed and grey literature, are theoretical reflections. Only 3 of the 10 included studies conduct interviews or focus groups. The thought-provoking fact is that none of these studies actively involve PLWD, potentially contributing to epistemic injustice. The studies included talked a lot *about them* but did not talk *with them*. From the included resources construct descriptors did not emerge. The studies offered many suggestions about the situations in which epistemic injustice can be observed but they did not identify specific indicators. This could give rise to a new goal in future research. All the included sources have been published in the past ten years, mostly in Western Europe. This finding may highlight the need to explore the phenomenon in other geographical areas as well, to detect its presence and explore potential differences. None of the included studies suggest possible solutions and interventions to decrease the presence of epistemic injustice.

## Strengths and limitations

To the best of our knowledge, this is the first scoping review that maps the findings of present sources on the topic of epistemic injustice in relation to PLWD. A scoping review was chosen given the innovativeness of the topic and the expected small number of sources present. One of the strengths of this work is certainly that it sheds light on an under-explored topic. Among the limitations of the paper, we can mention the constraints on the search for grey literature. It was conducted through consultation with the research team, a snowball process, and a Librarian, which may have resulted in some relevant records missing. This scoping included both peer-reviewed and grey literature, but no significant differences between them were found in the results. The only detectable difference was found between the study by Matthews (2016) and the chapter by Capstick et al. (2015) where a opposite views are offered of film as a training and information tool on dementia care.

The topic of epistemic injustice in PLWD in the included studies involves and has been addressed by different disciplines, among which social sciences, philosophy, and psychology. This contributed to the significant differences in how the topic was approached. A quality assessment was not carried out for this scoping review in line with available guidelines (Tricco et al., 2018); thus, it is important to acknowledge that the heterogeneity in designs and methodologies employed in the included studies may limit the conclusiveness of our findings regarding impact. Additionally, the inclusion of records in English language only may limit the representativeness of our findings.

## Conclusions

PLWD experience epistemic injustice in various ways, primarily through exclusion from academic research due to communication difficulties and challenges in obtaining informed consent. Studies highlight that PLWD are often excluded not because they are incapable of participating, but because researchers and ethical committees face difficulties in effectively communicating with them. Additionally, even when PLWD are allowed to speak, their voices are frequently not believed or trusted, which further perpetuates epistemic injustice. The presence of epistemic injustice severely impacts PLWD by limiting their autonomy and voice. The dominant societal framing of dementia often restricts their ability to make decisions, particularly in contexts like advance directives. This marginalization in decision-making and research participation reinforces negative perceptions and reduces opportunities for PLWD to be heard and believed. Furthermore, the absence of active engagement with PLWD in research perpetuates this injustice, creating a cycle of exclusion and underestimation of their experiences.

## Supplementary Material

**Supplementary Information** The online version contains supplementary material available at https://doi.org/10.1007/s12144-025-08519-y.

Supplementary Material

## Figures and Tables

**Table 1:** Search string utilized in PubMed

(("Social Stigma"[MeSH Terms] OR "Ageism"[MeSH Terms] OR "Stereotyping"[MeSH Terms] OR ("epistemic injustice"[Title/>Abstract] OR "testimonial injustice"[Title/Abstract] OR "hermeneutical injustice"[Title/Abstract] OR "contributory injustice"[Title/Abstract] OR "stereotyp*"[Title/Abstract] OR "stigma*"[Title/Abstract] OR "Ageism"[Title/Abstract]) and ("dementia"[Title/Abstract] OR "alzheimer"[Title/Abstract] OR"cognitive decline"[Title/Abstract] OR "dementia"[MeSH Terms])) AND (english[Filter]

**Table 2:** Sources included from peer-reviewed literature

Authors/Year of Publication	Country	Study design and methodology	Aim	Key results
Groot et al., 2023	Netherlands	Narrative and dialogical methodology: three case studies with academic researchers	To demonstrate: (1) the importance of attending the non-verbal in order to prevent epistemic injustice in research (2) how a case-study approach and discussing ethical dilemmas with peers may help to unpack some of the ethical tensions that the researchers experience	Need to evaluate the appropriateness of research’s approach, balancing rational and intuitive forms of interaction and interpretation Importance of participatory and collaborative working between colleagues
Halonen et al., 2024	Finland	Theoretical reflections	To shed light on what enables or prevents PLWD from participating in research and how this is connected to epistemic injustice	Necessity by researchers to be more confident in inclusion of PLWD – regardless of stage of dementia Necessity to adequate methods and informed consent to population (i.e. with ongoing consent)
Jongsma et al., 2017	Germany	Qualitative study. Interviews structured and semi-structured to representativeness, members and policy makers of Patient’s Organizations (POs) in both fields of dementia and autism	To analyse how the voices of affected people are valued in POs and whether the inclusion or exclusion of voices is based on problematic prejudices or other types of injustice	Persistent stereotypes hamper the inclusion of affected members Being affected causes distrust in having the ‘capacity to know’
Matthews, 2016	Australia and Scotland	8 interviews and a focus group discussion with staff members in dementia care	To investigate the role that media might play in learning to listen differently in dementia care	Film in dementia care education can help to emphasize with PLWD, present and validate the PLWD’s point of view depending also on characteristics of care workers
Price & Hill, 2021	USA	Theoretical reflections	To formulate an extended self-stigmatising model to explain the Alzheimer Disease’s experience	Epistemic injustice as antecedent of spiral of silence and social death and consequence of low self-esteem and self-efficacy
Spencer, 2023	UK	Theorical and philosophical analysis	To extend the definition of testimonial injustice and include all communicative practices (verbal or non-verbal); to encompass the epistemic harms inflicted upon some of the most marginalised in our society	Importance to develops a communicative sensibility (over testimonial sensibility)
Vulliermet & Kenis, 2024	Belgium	Theoretical reflections	To reflect about the problematicity of the definition of dementia in the society and his implications in decisions and discussions about advance directives	The current framing of dementia stimulates bias and stigmatization towards PLWD and impact also on discussions about advance directives
Young et al., 2019	Australia and Canada	Theoretical reflections	To explore (1) injustices related to involvement of persons with dementia in the construction of knowledge (2) relationships between stigma and epistemic injustice associated with the lived experiences of PLWD	Epistemic injustice occurs when stereotypes of the deflated credibility of PLWD are internalised Stereotypes of deflated credibility take many forms and are perpetuated in various ways Credibility judgements may be affected by assumptions about future state Internalisation of stereotypes can cause ‘others’ to exclude PLWD from epistemic practices Internalisation of stereotypes (self-prejudice) can cause PLWD to withdraw themselves from epistemic practices

**Table 3:** Sources included from grey literature

Authors/Year of Publication	Country	Study design and methodology	Aim	Key results
Capstick et al., 2015	Germany	Book’s chapter	To analyse the representations of dementia in contemporary Western fiction film	Representations of PLWD in film contributes to perpetuating negative stereotypical views of them
Chattat et al., 2024	UK	Book’s chapter	To highlights how the presence of epistemic injustice in the context of dementia can influence social health	The presence of epistemic injustice can have consequences on diagnosis disclosure, care planning, decision-making involvement and marginalization of PLWD

## Data Availability

This study is a scoping review and did not generate or analyse any primary data. All sources of information are cited in the manuscript and are publicly available.
