# Supplementary material for: I’m still here and my opinion matters: a scoping review on the experience of epistemic injustice among people living with dementia
Source: Curr Psychol. Author manuscript; Available in PMC 2026 Jan 1. (PMC7618523; doi:10.1007/s12144-025-08519-y)
Supplement: Supplementary Material [file EMS211771-supplement-Supplementary_Material.docx]

**Preferred Reporting Items for Systematic reviews and Meta-Analyses extension for Scoping Reviews (PRISMA-ScR) Checklist**

| **SECTION** | **ITEM** | **PRISMA-ScR CHECKLIST ITEM** | **REPORTED ON PAGE #** |
| --- | --- | --- | --- |
| **TITLE** | | | |
| Title | 1 | I'm still here and my opinion matters: a scoping review on the experience of epistemic injustice among people living with dementia | Title page |
| **ABSTRACT** | | | |
| Structured summary | 2 | Epistemic injustice is a concept introduced by Fricker. It refers to wrongs done to individuals in their capacity as knowers, often due to prejudice or stereotypes. People living with dementia (PLWD) are particularly vulnerable to epistemic injustice due to cognitive, emotional and social aspects related to their condition and this could negatively affect their quality of life. This scoping review aims to map the available evidence on how epistemic injustice can influence the experience of PLWD. By adopting the PRISMA and Joanna Briggs Institute guidance for scoping reviews, we included peer-reviewed and grey literature in English that describe the relation between the presence of epistemic injustice (concept) and the experience of PLWD (population) across different geographical and cultural contexts (context). Searches in academic databases (Web of Science, Proquest, PubMed, Scopus and EbscoHost) and among grey literature (OpenAlex and AlmaStart Discovery Tool) were conducted in November 2024. Two independent reviewers screened abstracts and full texts. A thematic analysis of the results was carried out. We included 10 studies, of which 7 from database searches, 2 from grey literature and 1 from reference lists of included studies. There was high methodological heterogeneity but most of the included studies were theoretical reflections. PLWD can experience epistemic injustice, often being excluded from academic research due to communication challenges and difficulties in obtaining informed consent. Even when included, both in research and clinical practice, their voices are often not valued, limiting their participation in decisions like advance directives, reinforcing negative stereotypes. | 1 |
| **INTRODUCTION** | | | |
| Rationale | 3 | To effectively address our specific research questions, we adopted a scoping review as our chosen method of evidence synthesis. Scoping reviews are particularly suitable for mapping the existing literature, identifying gaps in knowledge, and clarifying concepts (Munn et al., 2018). By utilising this method, we were able to maintain a rigorous and transparent process for searching and synthesising evidence. | 5 |
| Objectives | 4 | The general objective of this review is to scope a recent body of literature, identify the types of available evidence on the impact of epistemic injustice in people living with dementia and to analyse knowledge gaps on this topic in according to guidance for authors when choosing between a systematic or scoping review approach (Munn et al., 2018).  Specific questions include:   1. How do people living with dementia experience epistemic injustice? 2. What is the impact of epistemic injustice on people living with dementia | 4 |
| **METHODS** | | | |
| Protocol and registration | 5 | A scoping review protocol was developed in November 2024 and is available on the Open Science Framework (<https://osf.io/vd3aj/>). | 5 |
| Eligibility criteria | 6 | Sources of evidence were assessed for inclusion based on the JBI’s criteria of Population, Concept and Context (Peters et al., 2020).  ***Population***  We included sources that examined the experiences of people living with dementia (PLWD) across various stages of the condition, as well as studies that compared these experiences with those of other clinical populations, such as individuals with psychiatric diagnoses or those with physical or cognitive disabilities. There was no specific inclusion criteria based on factors like age, gender, or other demographic or qualifying characteristics. The only studies we excluded were those that did not specifically address the experiences of PLWD, ensuring our focus remained on this group and their perspectives.  ***Concept***  The main concept explored in this scoping review is epistemic injustice, a term first introduced by philosopher Miranda Fricker in 2007. Epistemic injustice refers to the ways in which biases, prejudices, and stereotypes can affect the way individuals are perceived in terms of their credibility and ability to contribute to knowledge. This unequal distribution of credibility often results in certain groups being marginalized or excluded from the process of knowledge construction and sharing (Young et al., 2019). In her work, Fricker identified two forms of epistemic injustice: testimonial injustice, which occurs when a person’s word is given less weight due to prejudice, and hermeneutic injustice, which arises when there is a gap in collective understanding or interpretative resources, leaving certain experiences or perspectives underrepresented or misrepresented. In this review, we specifically focused on studies that address one or both forms of epistemic injustice, aiming to shed light on the ways in which such injustices manifest and impact our population of interest.  ***Context***  We did not impose any exclusion criteria related to the context in which the research took place. Our primary interest was in mapping the different settings where research in this field has been carried out. By doing so, we aimed to identify any potential gaps, whether geographical, socio-cultural, or related to specific contexts, that might exist in the existing body of literature. This approach allowed us to gain a broader understanding of where research has been focused, while also highlighting areas that may be underrepresented or overlooked, offering insights into where further studies could be particularly valuable. | 5 |
| Information sources* | 7 | Our searches included peer-reviewed and grey literature in five electronic databases: PubMed, Web of Science, ProQuest Central, Scopus and EBSCOhost. The following databases were included in EBSCOhost: APA PsycInfo, APA PsycArticles, CINAHL Complete and Psychology and Behavioral Sciences Collection. Other studies were included by searching the bibliographies of the sources previously included. | 6 |
| Search | 8 | Search string utilized in PubMed.  (("Social Stigma"[MeSH Terms] OR "Ageism"[MeSH Terms] OR "Stereotyping"[MeSH Terms] OR ("epistemic injustice"[Title/Abstract] OR "testimonial injustice"[Title/Abstract] OR "hermeneutical injustice"[Title/Abstract] OR "contributory injustice"[Title/Abstract] OR "stereotyp*"[Title/Abstract] OR "stigma*"[Title/Abstract] OR "Ageism"[Title/Abstract]))  AND  ("dementia"[Title/Abstract] OR "alzheimer"[Title/Abstract] OR "cognitive decline"[Title/Abstract] OR "dementia"[MeSH Terms])) AND (english[Filter]) | 6 |
| Selection of sources of evidence | 9 | We retrieved a total of 6929 records from the online databases. We removed 2930 duplicates, leaving 3999 records to screen in stage 1. Of these, 3983 were excluded at stage 1, whereas 16 records moved to stage 2 (full-text screening). At this stage, 9 records were excluded because they did not meet inclusion criteria. Seven of these were excluded due to lack of focus on epistemic injustice (concept) and 2 because they did not focus on epistemic injustice (concept) and did not talk about people living with dementia (population). A total of 7 records from the database search were included in the review.  Beyond the database searches, we identified 5 records from the grey literature from websites and 4 records from the reference lists of included studies. Of these additional 9 records, 6 were excluded during the full text screening: 4 because they did not talk about epistemic injustice and 2 because they did not talk about people living with dementia.  In summary, a total of 10 records were included, of which 7 were from database searches, 2 from grey literature and 1 from reference lists of included studies. A PRISMA flow diagram outlining the screening and selection process is included in Fig. 1. | 8 |
| Data charting process | 10 | Once the searches were completed, the identified records were imported into the Rayyan software (Ouzzani et al., 2016) for removal of duplicates and abstract screening. We employed a three-step screening process with initial remotion of duplicates and a consequent assessment of title and abstract in Rayyan, followed by full-text screening carried out independently by two authors (L.C. and M.B.). Any discrepancies were resolved in discussion with a third reviewer (R.C.) or with other members of the research team. The final included studies were entered in an Excel spreadsheet, documenting key information including authors, year of publication, country, methods, participants, aims and results. | 6 |
| Data items | 11 | The final included studies were entered in an Excel spreadsheet, documenting key information including authors, year of publication, country, methods, participants, aims and results. | 6 |
| Critical appraisal of individual sources of evidence | 12 | Given the inclusion of both peer-reviewed and grey literature, and of different publication types or study designs, we did not carry out a structured critical appraisal of the evidence. This is in line with JBI’s guidance for scoping review, which indicates that quality assessment of individual sources or of meta-bias may not be possible in scoping reviews given the diversity of sources included and the broad aim of mapping the available literature for an understudied topic (Peters et al., 2020). | 6 |
| Synthesis of results | 13 | A thematic analysis was carried out to outline trends in publications’ key study characteristics based on data extraction. The findings were analysed by identifying thematic areas in the included studies, focusing on the context where the presence of epistemic injustice in people living with dementia can be observed. This categorisation was deemed more meaningful than organising on type of methodology used, as the high heterogeneity across the studies included. The aim of our main analysis was to identify how people living with dementia experience epistemic injustice (question 1) and what is its impact on their lives (question 2). | 7 |
| **RESULTS** | | | |
| Selection of sources of evidence | 14 | We retrieved a total of 6929 records from the online databases. We removed 2930 duplicates, leaving 3999 records to screen in stage 1. Of these, 3983 were excluded at stage 1, whereas 16 records moved to stage 2 (full-text screening). At this stage, 9 records were excluded because they did not meet inclusion criteria. Seven of these were excluded due to lack of focus on epistemic injustice (concept) and 2 because they did not focus on epistemic injustice (concept) and did not talk about people living with dementia (population). A total of 7 records from the database search were included in the review.  Beyond the database searches, we identified 5 records from the grey literature from websites and 4 records from the reference lists of included studies. Of these additional 9 records, 6 were excluded during the full text screening: 4 because they did not talk about epistemic injustice and 2 because they did not talk about people living with dementia.  In summary, a total of 10 records were included, of which 7 were from database searches, 2 from grey literature and 1 from reference lists of included studies. A PRISMA flow diagram outlining the screening and selection process is included in Fig. 1.  Give numbers of sources of evidence screened, assessed for eligibility, and included in the review, with reasons for exclusions at each stage, ideally using a flow diagram. | 8 |
| Characteristics of sources of evidence | 15 | The main characteristics of the records included in the review are presented in Tab. 2 (peer-reviewed literature) and Tab. 3 (grey literature). All the records included were published in the last 10 years. In terms of country distribution, 7 out of 10 studies were conducted in Europe, of which 2 in the United Kingdom, 2 in Germany, 1 in Belgium, 1 in Finland and 1 in the Netherlands. One study was conducted in the USA. Two studies were conducted in multiple countries: 1 between Australia and Canada and 1 between Australia and Scotland.  Regarding study methodologies, all the included studies used a qualitative method (n = 10). Most of the included studies (n = 7) are theoretical reflections, while the remaining are case studies (n = 1), interviews (n = 1) or interviews mixed with focus groups (n = 1). Publication types included empirical peer-reviewed articles (n = 8) and book chapters (n = 2).  Often the studies included were the result of collaboration between researchers from different disciplines (e.g., social health, psychology, philosophy). This contributed to the great methodological variety with which the topic was treated. | 9 |
| Critical appraisal within sources of evidence | 16 | Not applicable | Click here to enter text. |
| Results of individual sources of evidence | 17 | Peer-reviewed literature (authors and key results)   1. **Groot et al., 2023.**   Need to evaluate the appropriateness of research’s approach, balancing rational and intuitive forms of interaction and interpretation. Importance of participatory and collaborative working between colleagues   1. **Halonen et al., 2024.**   Necessity by researchers to be more confident in inclusion of PLWD – regardless of stage of dementia. Necessity to adequate methods and informed consent to population (i.e. with ongoing consent)   1. **Jongsma et al., 2017**   Persistent stereotypes hamper the inclusion of affected members. Being affected causes distrust in having the ‘capacity to know’   1. **Matthews et al., 2016**   Film in dementia care education can help to emphasize with PLWD, present and validate the PLWD’s point of view depending also on characteristics of care workers   1. **Price & Hill, 2021**   Epistemic injustice as antecedent of spiral of silence and social death and consequence of low self-esteem and self-efficacy   1. **Spencer, 2023**   Importance to develops a communicative sensibility (over testimonial sensibility)   1. **Vulliermet & Kenis, 2024**   The current framing of dementia stimulates bias and stigmatization towards PLWD and impact also on discussions about advance directives   1. **Young et al., 2019**   Epistemic injustice occurs when stereotypes of the deflated credibility of PLWD are internalised. Stereotypes of deflated credibility take many forms and are perpetuated in various ways. Credibility judgements may be affected by assumptions about future state. Internalisation of stereotypes can cause ‘others’ to exclude PLWD from epistemic practices. Internalisation of stereotypes (self-prejudice) can cause PLWD to withdraw themselves from epistemic practices  Grey literature (authors and key results)   1. **Capstick et al. (2015)**   Representations of PLWD in film contributes to perpetuating negative stereotypical views of them   1. **Chattat et al. (2024)**   The presence of epistemic injustice can have consequences on diagnosis disclosure, care planning, decision-making involvement and marginalization of PLWD | 10-14 |
| Synthesis of results | 18 | A thematic analysis was carried out to outline trends in publications’ key study characteristics based on data extraction. The findings were analysed by identifying thematic areas in the included studies, focusing on the context where the presence of epistemic injustice in people living with dementia can be observed. This categorisation was deemed more meaningful than organising on type of methodology used, as the high heterogeneity across the studies included. The aim of our main analysis was to identify how people living with dementia experience epistemic injustice (question 1) and what is its impact on their lives (question 2). | 7 |
| **DISCUSSION** | | | |
| Summary of evidence | 19 | The studies included in this scoping review introduce a great variety of context where it is possible to observe the occurrence of epistemic injustice. Four studies (Price & Hill, 2021; Spencer, 2023; Young et al., 2019; Chattat et al., 2024) include epistemic injustice within existing theoretical models (Price & Hill, 2021) or reflect on possible practical implications for PLWD. Two studies (Groot et al., 2023; Halonen et al., 2024) have further examined the reasons why PLWD tend to be excluded from participation in academical research. This seems to be associated to a difficult played by researchers and by ethical committee. In the study of Groot et al. (2023) researchers outlined their difficult to communicate and understanding PLWD; while in the study of Halonen et al. (2024) is highlight the difficult played by ethical committee in include also people who are not always able to give their informed consent at participation in research. Both studies mentioned above place the focus on difficulties tried by researchers, emphasizing how people with dementia are excluded from participating in academical research not because they are not able to participate, but because of the difficulties faced by the researchers. One study (Jongsma et al., 2017) interviewed representatives, members and policy makers of Patient’s Organization (POs) both in the field of dementia and autism. This study reveals that being heard is not sufficient. Even when individuals with dementia are given the opportunity to speak, their accounts are not necessarily acknowledged as valid contributions or afforded epistemic credibility. This allows us to say that to decrease the presence of epistemic injustice, it is not enough to listen to people with dementia but also to recognise the value of their testimonies. Alzheimer Europe emphasizes the significance of engaging individuals with dementia in research not only as study participants but also through active roles within Patient and Public Involvement (PPI). This includes contributing to the development of research ideas, offering input and guidance to researchers, participating in consultations, and taking part directly in the conduct of research activities (Gove et al., 2017). Evidence indicates that many people living with dementia can make valuable contributions to research on topics that directly impact their lives (Gove et al., 2017). Their involvement in PPI can be facilitated through diverse methodological approaches, including qualitative, quantitative, mixed-methods, co-production, co-research, and other participatory strategies (Gove et al., 2017). A variety of data collection methods may be used in this context, such as interviews, focus groups, questionnaires, and surveys (Gove et al., 2017). One study (Vulliermet & Kenis, 2024) analyses the implications of the presence of epistemic injustice in advance directives and ethical questions. The dominant negative and destructive framing of dementia denies at PLWD the right to take different decisions from those made before having a diagnosis of dementia. Two sources (Matthews, 2016; Capstick et al., 2015) investigate the impact of media in change the representation of dementia; the authors state that media can have a great impact on how the society see dementia and affected people, so it can be considerate a tool to change the negative representation of the disease, considering also the point of view of people with dementia. The variety of context of application of the concept can be considerate a great richness of this paper because it allows us to see in how many contexts it is possible to observe the presence of epistemic injustice. | 19 |
| Limitations | 20 | To the best of our knowledge, this is the first scoping review that maps the findings of present sources on the topic of epistemic injustice in relation to people living with dementia. A scoping review was chosen given the innovativeness of the topic and the expected small number of sources present. One of the strengths of this work is certainly that it sheds light on an under-explored topic. Among the limitations of the paper, we can find the search for grey literature. It was conducted through consultation with the research team, a snowball process and a Librarian, which may have resulted in some relevant records missing. This scoping included both peer-reviewed and grey literature, but no significant differences were found in the results between them. The only detectable difference is between the study by Matthews (2016) and the chapter by Capstick et al. (2015) where a different view is given of film as a training and information tool on dementia care. The topic of epistemic injustice in people with dementia in the included studies has been addressed by different disciplines: social sciences, philosophy and psychology. This contributed to the great differences on the ways in which the topic was approached. A quality assessment was not carried out for this scoping review in line with available guidelines; thus, it is important to acknowledge that the heterogeneity in designs and methodologies employed in the included studies may limit the conclusiveness of our findings regarding impact. Additionally, the inclusion of records in English language only may limit the representativeness of our findings. | 22 |
| Conclusions | 21 | People living with dementia (PLWD) experience epistemic injustice in various ways, primarily through exclusion from academic research due to communication difficulties and challenges in obtaining informed consent. Studies highlight that PLWD are often not excluded because they are incapable of participating, but because researchers and ethical committees face difficulties in effectively communicating with them. Additionally, even when PLWD are allowed to speak, their voices are frequently not believed or trusted, which further perpetuates epistemic injustice. The presence of epistemic injustice severely impacts PLWD by limiting their autonomy and voice. The dominant societal framing of dementia often restricts their ability to make decisions, particularly in contexts like advance directives. This marginalization in decision-making and research participation reinforces negative perceptions and reduces opportunities for PLWD to be heard and believed. Furthermore, the absence of active engagement with PLWD in research perpetuates this injustice, creating a cycle of exclusion and undervaluation of their experiences. | 23 |
| **FUNDING** | | | |
| Funding | 22 | This paper is supported by project EPIC that was generously funded by Wellcome. Grant [226603/z/22/z], 'EPIC: Epistemic Injustice in Health Care'. | Title page |

JBI = Joanna Briggs Institute; PRISMA-ScR = Preferred Reporting Items for Systematic reviews and Meta-Analyses extension for Scoping Reviews.

* Where *sources of evidence* (see second footnote) are compiled from, such as bibliographic databases, social media platforms, and Web sites.

† A more inclusive/heterogeneous term used to account for the different types of evidence or data sources (e.g., quantitative and/or qualitative research, expert opinion, and policy documents) that may be eligible in a scoping review as opposed to only studies. This is not to be confused with *information sources* (see first footnote).

‡ The frameworks by Arksey and O’Malley (6) and Levac and colleagues (7) and the JBI guidance (4, 5) refer to the process of data extraction in a scoping review as data charting*.*

§ The process of systematically examining research evidence to assess its validity, results, and relevance before using it to inform a decision. This term is used for items 12 and 19 instead of "risk of bias" (which is more applicable to systematic reviews of interventions) to include and acknowledge the various sources of evidence that may be used in a scoping review (e.g., quantitative and/or qualitative research, expert opinion, and policy document).

*From:* Tricco AC, Lillie E, Zarin W, O'Brien KK, Colquhoun H, Levac D, et al. PRISMA Extension for Scoping Reviews (PRISMAScR): Checklist and Explanation. Ann Intern Med. 2018;169:467–473. [doi: 10.7326/M18-0850](http://annals.org/aim/fullarticle/2700389/prisma-extension-scoping-reviews-prisma-scr-checklist-explanation).
